# *d*‐Wave Fermi Surface Instability in the Nematic Phase of Two Monolayer FeSe/SrTiO_3_

**DOI:** 10.1002/advs.202516394

**Published:** 2025-11-28

**Authors:** C. Y. Tang, X.‐L. Peng, Y.‐H. Yuan, P. Zhang, G.‐N. Phan, S.‐Y. Gao, Y.‐B. Huang, L.‐Y. Kong, T. Qian, W. Li, Q.‐K. Xue, Z.‐Q. Wang, K. Jiang, Y.‐J. Sun, H. Ding

**Affiliations:** ^1^ Beijing National Laboratory for Condensed Matter Physics and Institute of Physics Chinese Academy of Sciences Beijing 100190 China; ^2^ School of Physics University of Chinese Academy of Sciences Beijing 100190 China; ^3^ State Key Laboratory of Surface Physics and Department of Physics Fudan University Shanghai 200438 China; ^4^ State Key Laboratory of Low‐Dimensional Quantum Physics Department of Physics Tsinghua University Beijing 100084 China; ^5^ School of Physics Nanjing University Beijing 210093 China; ^6^ Shanghai Advanced Research Institute Chinese Academy of Sciences Shanghai 201204 China; ^7^ Department of Physics and Guangdong Basic Research Center of Excellence for Quantum Science Southern University of Science and Technology (SUSTech) Shenzhen 518055 China; ^8^ Department of Physics Boston College Chestnut Hill MA 02467 USA; ^9^ Quantum Science Center of Guangdong Hong Kong‐Macao Greater Bay Area Shenzhen Guangdong 518045 China; ^10^ Tsung‐Dao Lee Institute & School of Physics and Astronomy Shanghai Jiao Tong University Shanghai 200240 China

**Keywords:** *d*‐wave Fermi surface instability, iron‐based superconductor, nematicity, thin films

## Abstract

Nematicity, where electrons break rotational symmetry while preserving translational symmetry, is ubiquitous in strongly correlated quantum matters, including high‐*T*_c_ cuprates and iron‐based superconductors. A central question in nematicity is whether it is driven by Fermi surface instability in momentum space or orbital order (polarization) in real space, especially as nematicity intertwines with superconductivity. FeSe/SrTiO_3_ (STO), where nematicity occurs without long‐range magnetic order, is an ideal platform for studying the nature and origin of the electronic nematicity. Here, direct evidence of *d*‐wave nematic order in two monolayer FeSe/STO using angle‐resolved photoemission spectroscopy is presented, revealing a remarkable degeneracy of *d_xz_* and *d_yz_* bands at the Brillouin zone center, but a significant band separation at the zone corner. This momentum‐dependent nematicity demonstrates that nematicity in FeSe/STO originates from the *d*‐wave Fermi surface instability of the Pomeranchuk‐type, offering insights into the relationship between nematicity and superconductivity. The results establish 2D FeSe thin film as a powerful platform for investigating quantum physics under complex intertwinement.

## Graphical abstract

Angle‐resolved photoemission spectroscopy reveals a *d*‐wave nematic order in two‐monolayer FeSe/SrTiO_3_, showing degenerate *d_xz_*/*d_yz_* bands at Γ but a pronounced splitting at M. This momentum‐dependent anisotropy identifies the nematicity as a *d*‐wave Fermi surface (Pomeranchuk) instability, highlighting 2D FeSe as a model platform to explore the interplay between nematicity and superconductivity.

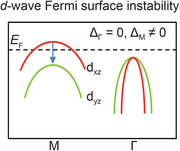

## 1 Introduction

Electronic nematicity, characterized by the breaking of lattice rotational symmetry while preserving translational symmetry in quantum electronic states, has garnered significant attention due to its prevalence in strongly correlated systems, including copper oxide superconductors,^[^ ^1^, ^2^ ^]^ iron‐based superconductors (FeSCs),^[^ ^3^, ^4^, ^5^, ^6^ ^]^ and twist bilayer graphene.^[^ ^7^ ^]^ The ubiquitous presence of nematicity and its intimate relationship with the superconducting regime in phase diagrams suggest that understanding nematicity is critical to unraveling the microscopic mechanisms of unconventional superconductivity.^[^ ^6^, ^7^, ^8^, ^9^, ^10^, ^11^, ^12^ ^]^ In this regard, FeSCs provide an ideal platform for studying electronic nematicity, due to their pronounced nematic phase transition, universality across diverse material families, and high tunability through doping and pressure. Among them, the FeSe/SrTiO_3_ (STO) system has an unprecedentedly high superconducting critical temperature (*T*_c_), and a nematic transition that occurs without the onset of long‐range magnetic order, unambiguously representing the cleanest demonstration of electronic nematicity. While nematicity in one monolayer (ML) system is suppressed by the high‐temperature superconducting transition, superconductivity does not take place and cannot even be proximitized to a 2 ML system, making 2 ML FeSe/STO an ideal platform for studying the nematic phase.

**Figure 1:**
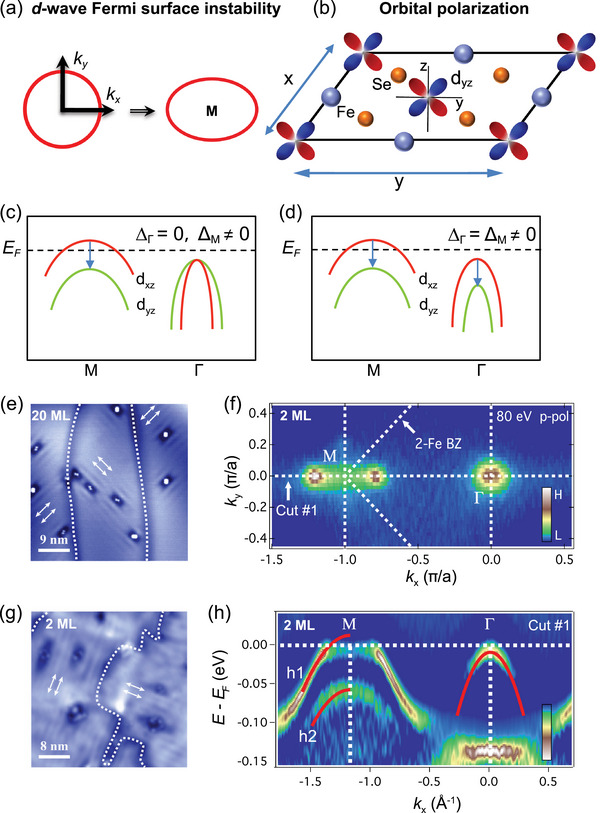
Two types of nematicity in FeSe thin films on SrTiO_3_ (STO). a) Schematic of *d*‐wave Fermi surface (FS) instability of the Pomeranchuk‐type induced deformation in the momentum space, and b) imbalance occupation in the *d_xz_*/*d_yz_* orbital induced orbital polarization in the real space, respectively. c) Schematic of *d_xz_*/*d_yz_* band degeneracy splitting at Γ and M driven by *d*‐wave FS instability, and d) orbital polarization, respectively. e) STM topographic image of a 20 monolayer (ML) FeSe film on STO (45 × 45 nm; *V* = 60 mV, *I* = 40 pA). The white arrows indicate the stripes with twofold symmetry. The direction of stripes rotates 90° when crossing the domain walls (the white dashed lines). f) The Fermi surface of 2 ML FeSe/STO measured by 80 eV *p‐*polarized photons at 20 K. The intensity has been integrated in the ±10 meV energy range. g) same as e but for a 2 ML FeSe film on STO (40 × 40 nm; *V* = 60 mV, *I* = 100 pA). h) Second‐derivative spectrum of the band structure of 2 ML FeSe/STO measured by 80 eV *p‐*polarized photons along the Γ‐M direction. The red lines represent the energy band near the Γ and M points. Bands *h1* and *h2* denote two hole‐like bands.

To understand the nematic order, the central question lies in addressing the driving mechanism of nematicity.^[^ ^13^ ^]^ Phenomenologically, there are two types of driving mechanisms of the nematic order in FeSe: the Fermi surface (FS) deformation of the Pomeranchuk‐type in momentum space^[^ ^9^, ^14^, ^15^, ^16^, ^17^, ^18^, ^19^, ^20^ ^]^ and orbital order (polarization) in real space arising from the occupation imbalance of degenerate atomic orbitals.^[^ ^21^, ^22^ ^]^ For the Pomeranchuk instability in Fermi liquids, quasiparticle interactions in the angular momentum *l* = 2 channel cause the FS to deform with a *d*‐wave form factor, resulting in rotational symmetry‐breaking FSs invariant under inversion in momentum space, as shown in **Figure** **1**a. The concept of a *d*‐wave Pomeranchuk instability was proposed theoretically and subsequently supported by experiments.^[^ ^14^, ^15^, ^16^, ^17^, ^18^, ^19^, ^20^ ^]^ On the other hand, it is also possible to generate nematicity from the real‐space perspective. Under C_4_ rotational symmetry, the rotation‐related *d_xz_* and *d_yz_* orbitals host equal occupation. When an onsite/local orbital‐dependent energy difference lifts the degeneracy and causes an imbalance in orbital occupation, it leads to an orbital polarization favoring the *d_yz_* orbital, as illustrated in Figure 1b. Regarding orbital‐driven nematicity, many possible orbital‐ordering terms have been proposed. Here, we mainly focus on the most established ferro‐orbital scenario, where unequal occupation of *d_xz_* and *d_yz_* orbitals induces uniform energy splitting. The consequences of the above two mechanisms are different. For the orbital polarization, it is local and homogeneous in real space, resulting in an isotropic degeneracy splitting of the energy levels of *d_xz_* and *d_yz_* orbitals in momentum space. Hence, an isotropic band separation (Δ_Γ_ = Δ_M_ ≠ 0) opens at both the Γ and M points, as shown in Figure 1d. For the Pomeranchuk nematic order, the symmetry breaking in the *l* = 2 angular momentum channel has lattice harmonics that are anisotropic in momentum space, being zero at the Γ point and maximum at the M point. Therefore, the *d*‐wave FS (Pomeranchuk) instability only generates maximum bands splitting at the M with Δ_M_ ≠  0 around which the FSs of FeSe/STO are located, as illustrated in Figure 1c, but leaves the bands at Γ degenerate with Δ_Γ  _= 0. Note that in different domains, the orbital characters of *d*_*xz*_ and *d*_*yz*_ swap, altering the band separation energy, as indicated in Figure 3e. In this work, we isolate the critical role of the *d*‐wave FS instability in the nematic phase of 2 ML FeSe/STO, and provide direct evidence that nematicity predominantly originates from the Pomeranchuk instability‐induced FS deformation in momentum space. Our work establishes FeSe thin films as an ideal platform for exploring profound quantum physics, including intertwined order, nematicity, and superconductivity.

## 2 Results

The nematic order emerges in FeSe thin films on STO substrates when the temperature decreases below the nematic transition temperature *T*_nem_,^[^ ^23^, ^24^ ^]^ and the nematicity enhances with decreasing layer thickness. As shown in Figure 1e of STM topography, stripes develop in the vicinity of impurities in the 20 ML FeSe film. Upon reducing the film thickness to 2 ML, the nematicity becomes more pronounced, exhibiting smectic‐like features^[^ ^25^ ^]^ as illustrated in Figure 1g. Here, the term “smectic‐like” refers to short‐range, unidirectional electronic modulations that emerge as fluctuations of an amplified nematic order, rather than a true smectic phase with long‐range translational symmetry breaking. The direction of these stripes changes 90° across the domain walls, signifying the breaking of the four‐fold rotational symmetry within each domain, providing direct evidence of electronic nematicity. Notably, stripes have not been observed in 1 ML FeSe/STO,^[^ ^26^ ^]^ likely due to the heavy electron doping and strong electron‐phonon coupling from the substrate, driving the material into the high‐*T*_c_ superconducting state.^[^ ^27^ ^]^ In contrast, the high‐*T*_c_ state does not persist or even proximitize to the second layer of FeSe, which is weakly coupled to the first layer and can be evaporated by low‐temperature annealing, akin to the second layer of epitaxially grown graphene.^[^ ^28^, ^29^, ^30^, ^31^ ^]^ The Fermi surface of 2 ML FeSe, as shown in Figure 1f, is consistent with previous results.^[^ ^11^, ^28^ ^]^ It should be noted that the hole‐like bands around the Γ point do not cross the Fermi level. The finite spectroscopic intensity observed near Γ is not from FS but from measurement resolution broadening. Figure 1h illustrates the band structure measured along the Γ‐M direction for 2 ML FeSe, with the hole‐like bands at Γ situated below the Fermi energy (*E*_F_), indicating that the FS of 2 ML FeSe is solely around the zone corner. The nematicity induces a band separation at the M point between the two hole‐like bands (see also Figure S1, Supporting Information), defined as Δ_M_, which mirrors the energy separation observed in detwinned FeSe single crystals.^[^ ^32^, ^33^, ^34^ ^]^ Notably, the band separation size is ≈70 meV, surpassing the ≈50 meV detected in bulk FeSe at 30 K.^[^ ^33^ ^]^

**Figure 2:**
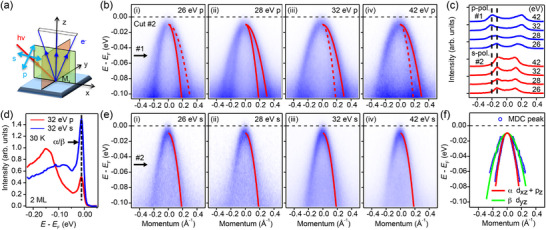
Degeneracy of the *d_xz_*/*d_yz_* orbitals at the Brillouin Zone center (Γ) in 2 ML FeSe on STO. a) Schematic experimental geometry for the ARPES measurements. b) Band structure measured by i) 26 eV, ii) 28 eV, iii) 32 eV, and iv) 42 eV *p‐*polarized photons along the Γ‐M direction. The red lines represent the fitting results of two hole‐like bands. Solid and dashed lines indicate the visible and invisible bands, respectively. c) Comparison of the momentum distribution curve (MDC) measured by different photon energies and polarizations at the energies indicated by #1 in (b) and #2 in e). The black dashed line indicates the peak position. d) Comparison of the energy distribution curve (EDC) at the Γ point measured by 32 eV *p‐* and *s‐*polarized photons. The black dashed line indicates the position of the *α* and *β* band tops. e) Similar to (b) but measured by *s‐*polarized photons. f) Summary of the band structure and orbital analysis around Γ. Blue circles represent the peak position of MDCs fitted by the Lorentz function. Two solid curves are the fitting results of the MDC peaks.

In angle‐resolved photoemission spectroscopy (ARPES) measurements, photons with different polarizations can excite electrons with distinct wavefunction symmetries within crystals.^[^ ^35^ ^]^ The experimental geometry defines the *p*‐ and *s*‐polarization, as shown in **Figure** **2**a. Odd (even) orbital with respect to the M_x_ mirror plane are selectively excited by *p*‐ (*s*‐) polarized photons. The band dispersion along the Γ ‐ M direction measured by *p*‐polarized photons is shown in Figure 2b. Two hole‐like bands *α* and *β* are clearly observed with different photon energies (*hv* = 26, 28, 32, and 42 eV, respectively). As the photon energy increases, the intensity of the *α* band decreases while that of the *β* band increases, attributed to the opposite photoemission cross sections of Fe 3*d* and Se 4*p* orbitals in the energy range of our experiments.^[^ ^36^, ^37^ ^]^ Therefore, we can infer that the *β* band has Fe *d*_*yz*_ orbital character and the *α* band partially comprises the Se *p_z_* orbital character. On the other hand, when the exciting photons are switched to *s*‐polarization, only the *α* band is observed, as shown in Figure 2e, confirming its *d*_*xz*_ orbital character.

Significantly, unlike in bulk FeSe, the energy separation between the *α* and *β* bands at the Γ point (Δ_Γ_) in 2 ML FeSe vanishes, as shown in Figure 2b,c. To elucidate the energy separation more accurately, we present the energy distribution curve (EDC) at the Γ point, using data collected with 32 eV *p*‐ and *s*‐polarized photons, as illustrated in Figure 2d. We choose this exciting energy because both energy bands are enhanced, as shown in Figure 2b(iii),e(iii). It is evident that these two bands are degenerate. Furthermore, to quantitatively determine the band dispersion, we extract the peak positions of the momentum distribution curves (MDCs) and fit the data points with a parabola function, as illustrated in Figure 2f. The fitting results reveal that the band tops of the *α* and *β* bands are located at 9.39 ± 0.31 and 9.83 ± 1.05 meV below the Fermi energy (*E*_F_), respectively. This further proves the degenerate nature of the *d* *_xz_* and *d* *_yz_* bands at the Γ point. On general grounds, there are typically two common origins for the band separation Δ_Γ _at the Γ point: orbital polarization and spin‐orbit coupling (SOC). However, these two mechanisms cannot cancel each other out since they are coupled to different scattering channels.^[^ ^9^ ^]^ Therefore, the absence of the nematic band separation of the *d*_*xz*_ and *d*_*yz*_ bands at the Γ point rules out the orbital polarization scenario. On the other hand, the significant energy separation around the M point manifests the anisotropic nature of nematicity in the momentum space in 2 ML FeSe, consistent with the *d*‐wave FS instability‐induced nematic order, given that the FSs only exist around the M point.

**Figure 3:**
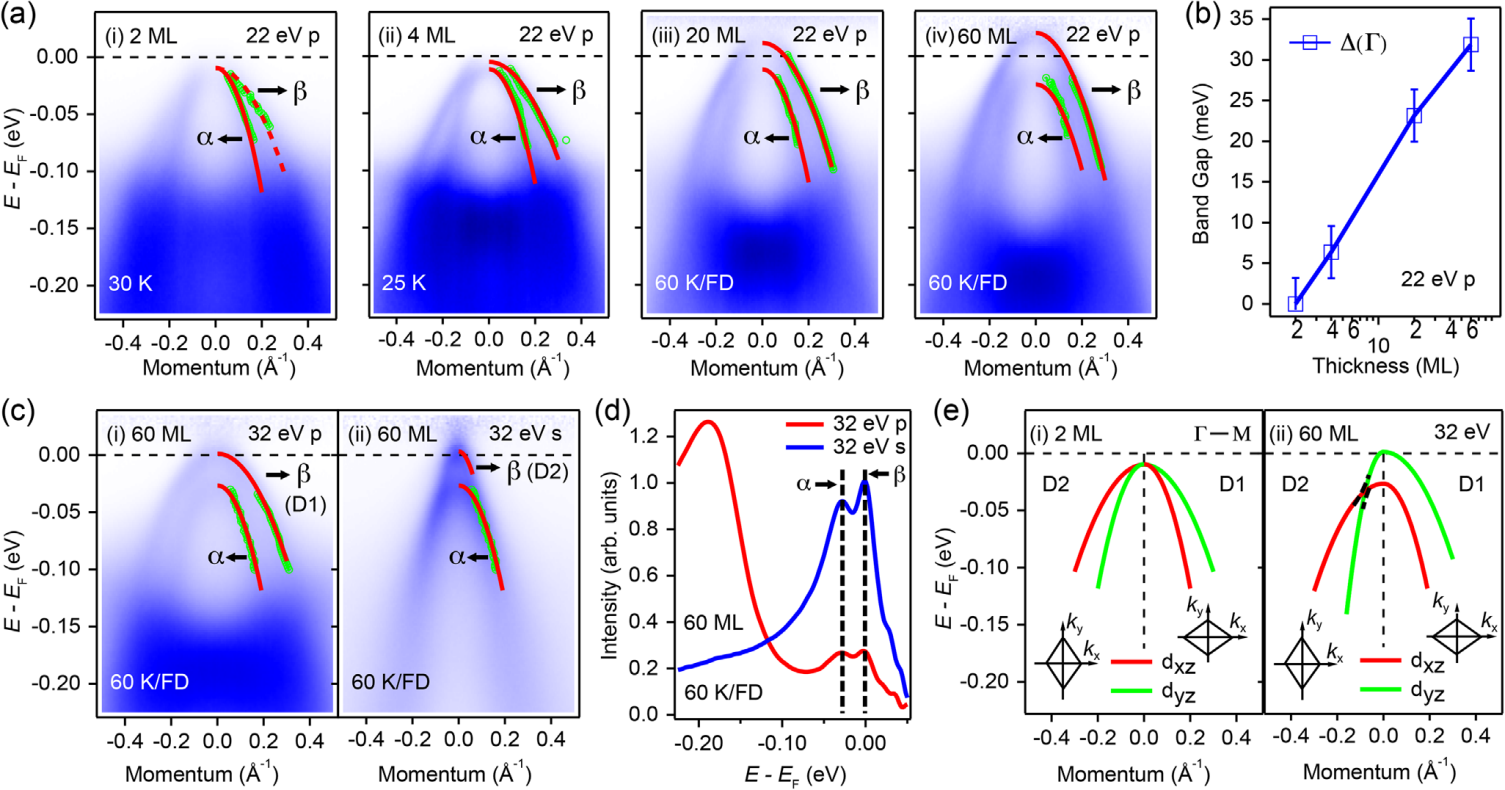
Thickness‐dependent *d_xz_*/*d_yz_* band separation and nematic order around the Γ point. a) Band structure of i) 2 ML, ii) 4 ML, iii) 20 ML, and iv) 60 ML FeSe measured by 22 eV *p‐*polarized photons along the Γ – M direction. The results of 20 and 60 ML are divided by the Fermi–Dirac distribution function convoluted by the resolution function to visualize the states above *E*_F_. Red lines represent the fitting results of band dispersion. b) Evolution of the band separation between *α* and *β* with film thickness. c) Band structure along the Γ – M direction of 60 ML FeSe measured by 32 eV i) *p‐* and ii) *s‐* polarized photons. The red lines represent the band dispersion of the *α* and *β* bands. D1 and D2 represent domain 1 and domain 2. d) Comparison of the energy distribution curve (EDC) at the Γ point of the data in (c). The black dashed lines indicate the positions of the *α* and *β* band tops. e) Schematic band structure around the Γ point of i) 2 ML and ii) 60 ML for two perpendicular domains. The *k_x_*‐axis is defined along the 2‐Fe Brillouin zone direction.

To further investigate the evolution of nematic order from an ultrathin film to bulk FeSe, we measured the band structure of FeSe films with thicknesses of 4, 20, and 60 MLs. **Figure** **3** represents the evolution of Δ_Γ_ as a function of film thickness. As shown in Figure 3a, the degeneracy of the two hole‐like bands is lifted in the 4 ML film, and the band separation increases systematically with film thickness. The energy difference Δ_Γ_, as shown in Figure 3b, gradually increases from 0 meV in the 2 ML film to 32 meV in the 60 ML FeSe film, a value comparable to that observed in bulk FeSe samples.^[^ ^32^, ^38^, ^39^ ^]^ This indicates that nematic splitting emerges in multilayer films at the Γ point.

In Figure 3c, employing 32 eV *p*‐ and *s*‐polarized photons, we observe two sets of hole‐like bands near the Γ point in 60 ML FeSe. It should be noted that the sample contains twined nematic domains, hence the two sets of energy bands belong to the *d_xz_* and *d_yz_* orbitals of different twin domains defined by their crystal coordinates, as shown in Figure 3e(ii). In contrast, we only observe one set of energy bands in the 2 ML film, due to the absence of nematic order at Γ, as illustrated in Figure 3e(i). Therefore, this observation is consistent with the *d*‐wave FS instability nature of the nematicity in the 2 ML film. The band separation (Δ_Γ_) of the 60 ML film measured by two different photon polarizations has the same value, as depicted in Figure 3d.

**Figure 4:**
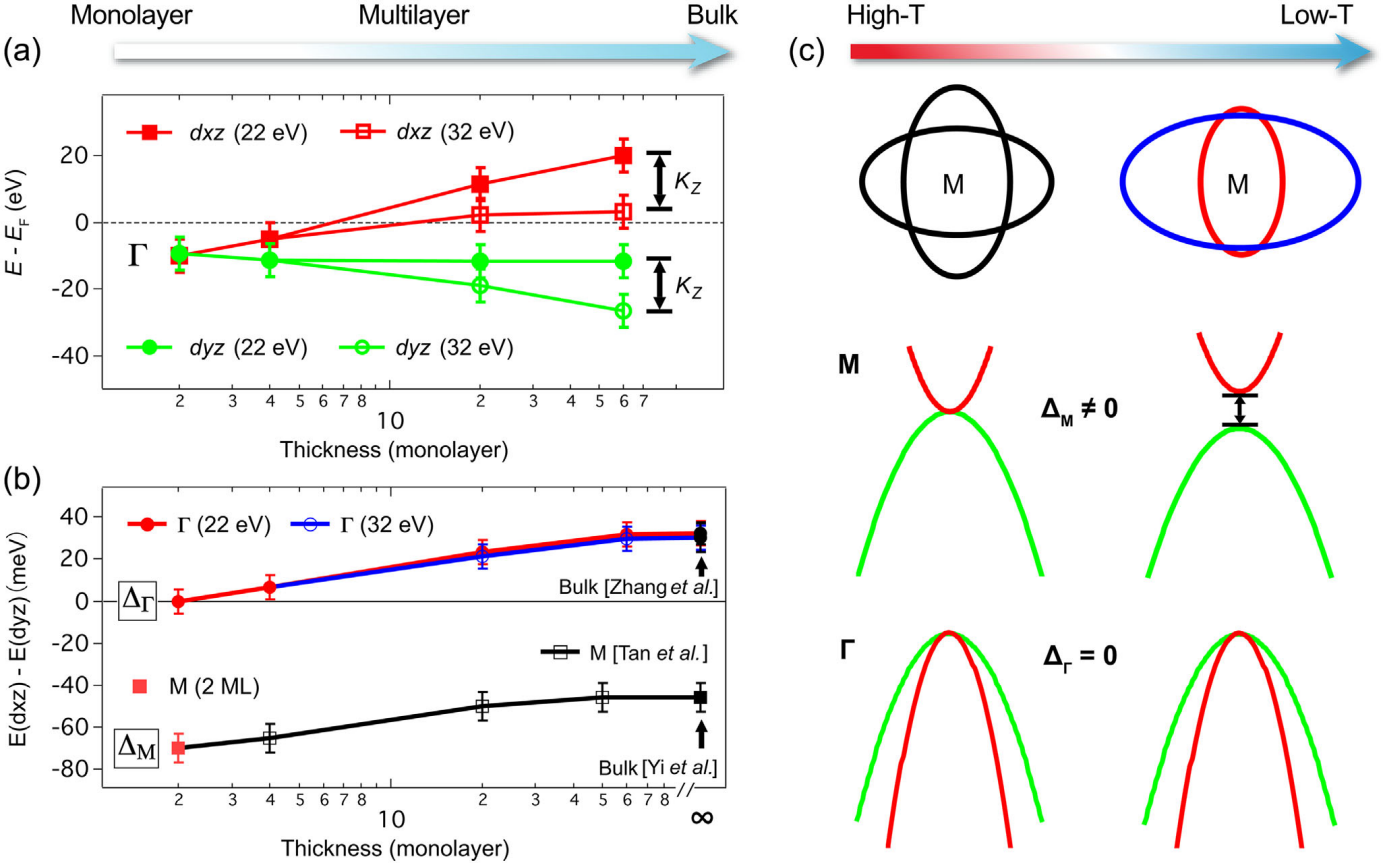
Summary of thickness‐dependent nematic order in FeSe films on STO. a) Summary of the thickness‐dependent binding energy of hole‐like bands at the Γ point and the corresponding orbital character measured by 22 and 32 eV photons. *k_z_* represents the variation of the band top with photon energy. b) Summary of the evolution of the onsite energy difference between the *d_xz_* and *d_yz_* orbitals at the Γ and M points with film thickness. The data of bulk FeSe are adapted from ref. [38] for the Γ point and ref. [33] for the M point. The data of the M point of multilayer FeSe are adapted from ref. [11]. c) Schematic of the *d*‐wave instability‐induced nematic distortion of FSs and the band structure near M and Γ point observed in 2 ML FeSe/STO, respectively.

To elucidate the band evolution more clearly, we summarize the binding energies of the *d_xz_* and *d_yz_* bands measured by 22 and 32 eV photons at the Γ point in **Figure** **4**a. In thicker films, the binding energy of each band varies when measured with different photon energies, indicative of band dispersion along *k* *_z_*. Despite the *k*_*z*_ dispersion, the nematicity‐induced band separation remains constant, as depicted in Figure 4b, where the results of Δ_Γ_ and Δ_M_ are presented as a function of film thickness. With increasing thickness, both Δ_Γ_ and Δ_M_ shift to higher values nearly in parallel, suggesting the presence of orbital polarization (Δ_Γ_ = Δ_M_ ≠ 0).

## 3 Discussion

In light of our results, the degeneracy of the *d_xz_*/*d_yz_* bands at Γ in 2 ML films indicates that the FS instability of the Pomeranchuk‐type is the primary driving force of the nematic transition. Furthermore, thinner films exhibit larger Δ_M_, smaller Δ_Γ_, and higher nematic transition temperatures.^[^ ^9^, ^28^, ^38^, ^39^, ^40^, ^41^ ^]^ We note that the strength of electronic anisotropy is determined primarily by the momentum region around the M point, where the nematic order parameter has its largest weight. This systematic trend can be naturally understood as a dimensionality‐driven amplification of the *d*‐wave Pomeranchuk instability. Reduced interlayer hopping and enhanced 2D confinement increase the *l* = 2 Landau‐interaction anisotropy, thereby strengthening the *d*‐wave nematic susceptibility. With increasing layer thickness, orbital polarization contributes significantly to the nematicity in FeSe. Accordingly, the momentum‐dependent sign‐changing nematic order observed in bulk FeSe likely comprises a predominant *d*‐wave nematic order and thickness‐dependent orbital polarization. Although a previous proposal suggested that three orbital orders could lead to accidental degeneracy,^[^ ^42^ ^]^ this hypothesis can be ruled out by the Γ point degeneracy at high temperatures, since distinct orbital orders should exhibit different temperature dependencies (see Figure S2, Supporting Information). Regarding the microscopic origin of the observed *d*‐wave FS instability, several theoretical scenarios have been proposed. One plausible mechanism is that quantum fluctuations induced by inter‐atomic Coulomb repulsion lead to a renormalized band structure where the van Hove singularity at M sits close to the Fermi level, facilitating the emergence of the *d*‐wave bond nematic order.^[^ ^9^ ^]^ Our observation of the nematic order with the *d*‐wave form (Δ_Γ  _= 0, Δ_M_ ≠  0) is consistent with the theoretical predictions. Alternatively, the *d*‐wave FS instability may be driven by spin fluctuations‐mediated Landau interaction in the *l* = 2 charge sector.^[^ ^6^, ^15^, ^43^, ^44^ ^]^ We emphasize that, however, our work is purely experimental and does not provide direct evidence for any specific microscopic mechanism. Our results therefore, provide empirical constraints on the theoretical models of electronic nematicity in FeSe, motivating further theoretical investigations into its microscopic origin.^[^ ^6^ ^]^

It is important to note that in 2 ML FeSe/STO, the *d_xz_* orbital does not hybridize with the *d_yz_* band at the Γ point, as confirmed by the absence of a hybridization gap and by the pronounced polarization dependence of the ARPES intensity. This lack of inter‐orbital hybridization induced by SOC significantly simplifies the low‐energy electronic structure, providing a uniquely clean platform for identifying the nematicity as a pure *d*‐wave Fermi surface (Pomeranchuk) instability. In other iron‐based superconductors, SOC has been extensively studied and discussed, it can induce a gap at the Γ point without breaking rotational symmetry.^[^ ^45^, ^46^, ^47^, ^48^ ^]^ In such systems, the FSs are strongly mixed, and the nematic order may arise from different microscopic routes. A natural explanation is that the SOC strength in 2 ML films is significantly reduced compared to other iron‐based materials. A previous ARPES study has indicated that the SOC strength is material‐dependent in iron‐based superconductors.^[^ ^45^ ^]^ Although the atomic SOC constant remains constant, the effective SOC strength in materials largely depends on the crystal field, electron correlations,^[^ ^49^ ^]^ and possibly spin fluctuations,^[^ ^50^, ^51^, ^52^, ^53^ ^]^ all of which may exhibit thickness‐dependent behavior. The SOC effect in FeSe thin films warrants further theoretical and experimental investigation.

We achieved an unprecedented understanding of the nematicity in FeSe, and unambiguously settled the debate on its origin by observing the *d*‐wave FS instability in 2 ML FeSe/STO, which exhibits the strongest nematicity. The *d*‐wave nematic order induces a distortion of FSs and lifts the degeneracy between the *d*_*xz*_ and *d*_*yz*_ orbitals around the M point, as shown in Figure 4c. Conversely, the *d*_*xz*_ and *d*_*yz*_ orbitals below the Fermi level are degenerated at the Γ point, ruling out the ferro‐orbital order scenario, as illustrated in Figure 4c. Further investigation of thickness‐dependent FeSe thin films reveals that the momentum‐dependent nematic order observed in bulk FeSe can be attributed to two primary components: the predominant *d*‐wave Pomeranchuk instability‐induced FS deformation and the thickness‐dependent orbital polarization. We emphasize that both mechanisms—the Pomeranchuk instability and orbital polarization—may coexist or interplay across different film thicknesses. Our results do not exclude the orbital polarization scenario, but rather highlight a potential interplay between multiple mechanisms.

Our findings establish a novel framework for probing the interplay between nematicity and high‐*T*_c_ superconductivity, imposing stringent constraints on candidate microscopic mechanisms of high‐*T*_c_ pairing. Beyond identifying pure *d*‐wave nematicity, the 2 ML FeSe/STO system exhibits intriguing emergent phenomena: the FS undergoes a dramatic topological reconstruction in 2 ML films, while the high‐*T*_c_ state observed in 1 ML FeSe/STO vanishes entirely in 2 ML, concomitant with the onset of a smectic‐like phase that amplifies nematic correlations. This sharp dichotomy between monolayer and bilayer behavior underscores the critical role of dimensionality in stabilizing competing quantum phases. Notably, recent work has identified nematic fluctuations‐mediated superconductivity in the FeSe_1‐_ *_x_* S*_x_* system.^[^ ^10^ ^]^ The isolated nematic phase can be suppressed by sulfur substitution at a quantum critical point, where nematic fluctuations are most pronounced. Introducing sulfur into our 2 ML FeSe/STO system presents a promising avenue to systematically tune nematic order, resolving whether superconductivity is enhanced, suppressed, or coexists with modified nematicity. Furthermore, many unconventional features of high‐*T*_c_ materials, including electronic nematicity, originate from their quasi‐2D nature, where quantum fluctuations are markedly enhanced. Our findings highlight that a 2D system not only provides fresh perspectives on the behavior of correlated quantum materials under complex intertwined orders, but also sheds light on fundamental physics in the 2D limit.

## 4 Experimental Section

### Thin‐Film Growth

High‐quality FeSe films for in situ ARPES measurements are grown on 0.7 wt% Nb‐doped SrTiO_3_ (001) substrates after degassing for 10 h at 600 °C, followed by annealing for 1.5 h at 950 °C in an ultrahigh vacuum (UHV) molecular beam epitaxy chamber. Substrates are kept at 310 °C during film growth. Fe (99.98%) and Se (99.999%) are co‐evaporated from Knudsen cells. The flux ratio of Fe to Se is 1:10, which is measured by a quart crystal balance. The growth rate determined by the Fe flux is fixed at 0.7 UC/min. During growth, sample quality is monitored using reflection high‐energy electron diffraction. After growth, the FeSe films are annealed at 370 °C for 10 h.

### ARPES and STM Measurements

The samples are transferred in situ to the ARPES chamber for measurements. The ARPES measurements are conducted at the BL‐09U “Dreamline” beamline of the Shanghai Synchrotron Radiation Facility (SSRF) using a VG DA30 electron analyzer under a UHV better than 5 × 10^−11^ torr. The energy resolution is set to ≈12 meV for the band structure and ≈16 meV for FS mapping, while the angular resolution is set to 0.2°. The spectra are recorded at 30 K unless specified otherwise. The scanning tunneling microscopy (STM) measurements are performed in a UHV STM (Unisoku) with a base temperature of 4.2 K. A polycrystalline PtIr STM tip is used and calibrated on Ag islands before the STM experiments.

## Conflict of Interest

The authors declare no conflict of interest.

## Supporting information



Supporting Information

## Data Availability

The data that support the findings of this study are available from the corresponding author upon reasonable request.
