# Supplementary material for: d‐Wave Fermi Surface Instability in the Nematic Phase of Two Monolayer FeSe/SrTiO3
Source: Adv Sci (Weinh). 2025 Nov 28;13(7):e16394. doi: 10.1002/advs.202516394 (PMC12866738; doi:10.1002/advs.202516394)
Supplement: Supplementary file 1 — Supporting Information [file ADVS-13-e16394-s001.docx]

**Supplementary Materials for**

*d*-wave Fermi Surface Instability in the Nematic Phase of Two Monolayer FeSe/SrTiO_3_

C. Y. Tang^1,2,3*^, X.-L. Peng^1,2*^, Y.-H. Yuan^4^, P. Zhang^5^, G.-N. Phan^1^, S.-Y. Gao^1,2^, Y.-B. Huang^6^, L.-Y. Kong^1^, T. Qian^1,2^, W. Li^4^, Q.-K. Xue^4,7^, Z.-Q. Wang^8^, K. Jiang^1†^, Y.-J. Sun^7,10,1†^ & H. Ding^1,9^

^1^Beijing National Laboratory for Condensed Matter Physics, and Institute of Physics, Chinese Academy of Sciences, Beijing 100190, China

*^2^School of Physics, University of Chinese Academy of Sciences, Beijing 100190, China*

*^3^State Key Laboratory of Surface Physics and Department of Physics, Fudan University, Shanghai 200438, China.*

*^4^State Key Laboratory of Low-Dimensional Quantum Physics, Department of Physics, Tsinghua University, Beijing 100084, China*

*^5^School of Physics, Nanjing University, Beijing 210093, China*

*^6^Shanghai Advanced Research Institute, Chinese Academy of Sciences, Shanghai 201204, China*

^7^Department of Physics and Guangdong Basic Research Center of Excellence for Quantum Science, Southern University of Science and Technology (SUSTech), Shenzhen 518055, China

*^8^Department of Physics, Boston College, Chestnut Hill, MA 02467, USA*

^9^Tsung-Dao Lee Institute & School of Physics and Astronomy, Shanghai Jiao Tong University, Shanghai 200240, China

^10^Quantum Science Center of Guangdong Hong Kong-Macao Greater Bay Area (Guangdong), Shenzhen 518045, China

^*^ These authors contributed equally to this work

^†^ Corresponding authors: jiangkun@iphy.ac.cn; sunyj@sustech.edu.cn

**This PDF file includes:**

Figures S1 to S2

**Figure S1.** Temperature dependence of nematic band separation in 2 ML FeSe/STO. a), d) Electronic structure along Γ - M direction detected by 80 eV *p*-polarized photons and the corresponding second derivative spectrum. The data is measured at 165 K. b), e) and c), f) Same as (a),(d) but measured at 130 K and 80 K, respectively. As the temperature decreases, the nematic band separation at the M point increases. This behavior is consistent with the results observed in bulk materials, affirming the existence of the nematic order in 2 ML FeSe/STO.

**Figure S2.** 𝛤 point *d_xz_*/*d_yz_* degeneracy at high temperature in 2 ML FeSe/STO. a), b) Band structure along Γ – M direction measured at 165 K by 32 eV *p-* and *s-* polarized photons, respectively. c) Comparison of energy distribution curve (EDC) at the Γ point measured by 32 eV *p-* and *s-*polarized photons. d), e), same as (a), (b), but divided by the Fermi–Dirac distribution function convoluted by the resolution function to highlight the states near and above the Fermi energy*.* f) Comparison of energy distribution curve (EDC) at the Γ point measured by 32 eV *p-* and *s-*polarized photons. The black dashed lines indicate the position of the *α* and *β* band tops.
